# The role of TREM2 in Alzheimer’s disease: from the perspective of Tau

**DOI:** 10.3389/fcell.2023.1280257

**Published:** 2023-11-08

**Authors:** Wendi Huang, Juan Huang, Nanqu Huang, Yong Luo

**Affiliations:** ^1^ Department of Neurology, Third Affiliated Hospital of Zunyi Medical University (The First People’s Hospital of Zunyi), Zunyi, China; ^2^ Key Laboratory of Basic Pharmacology of Ministry of Education and Joint International Research Lab of Ethnomedicine of Ministry of Education, Zunyi Medical University, Zunyi, Guizhou, China; ^3^ National Drug Clinical Trial Institution, Third Affiliated Hospital of Zunyi Medical University (The First People’s Hospital of Zunyi), Zunyi, Guizhou, China

**Keywords:** Alzheimer’s disease, triggering receptor expressed on myeloid cells 2, Tau, Tau pathology, soluble TREM2, microglia, neuroinflammation, phosphorylation

## Abstract

Triggering receptor expressed on myeloid cells 2 (TREM2), a pattern recognition receptor abundantly expressed on microglia, has been identified as one of the risk factors for Alzheimer’s disease (AD). Several studies have already demonstrated the relationship between TREM2 and Tau. TREM2 mutations and altered expression play an important role in Tau phosphorylation. Furthermore, the level of Tau phosphorylation is correlated with soluble TREM2 (sTREM2). However, in different stages of AD, TREM2 seems to have varying effects on Tau pathology. The explicit interaction between TREM2 and Tau, as well as how they affect AD pathology, remains unclear, and there is much evidence to the contrary that requires rational interpretation. Reviewing the dual roles of TREM2 in AD will help identify a more appropriate development strategy for targeting TREM2 to treat AD. Therefore, this review focuses on the interplay between Tau and TREM2 in relation to AD.

## 1 Introduction

Alzheimer’s disease (AD), a major cause of dementia, is characterized by the accumulation of amyloid-β peptide (Aβ), as well as the aggregation of hyperphosphorylated Tau protein (Wilson et al., 2023). The triggering receptor expressed on myeloid cells 2 (TREM2), a transmembrane receptor abundantly expressed on microglia, has been identified as one of the risk factors for AD (Hashioka et al., 2020). Studies have shown that mutations and polymorphisms of the *TREM2* gene are associated with a significant increase in the risk of AD (Jonsson et al., 2013; Cuyvers and Sleegers, 2016). In particular, the R47H variant is associated with a decrease in the number of receptors and loss of function (Guerreiro et al., 2013; Gussago et al., 2019; Sayed et al., 2021). Additionally, soluble TREM2 (sTREM2) is a potential AD biomarker (Brosseron et al., 2020) and can be detected in cerebrospinal fluid (CSF) in both healthy individuals and AD patients (Carmona et al., 2018), elevated sTREM2 levels noted in the CSF of AD patients (Yang et al., 2020). Moreover, apolipoprotein E (ApoE), TAR DNA-binding protein 43 (TDP-43), and other proteins closely associated with AD serve as ligands for TREM2 (Atagi et al., 2015; Xie et al., 2022).

Studies have found that the lack of TREM2 increases the hyperphosphorylation and aggregation of Tau and induces activation of microglia in the h-Tau mouse model as well (Bemiller et al., 2017). In P301S mice (an animal model of tau pathology), TREM2 overexpression promotes a corresponding increase in the protein levels of pro-inflammatory factors tumor necrosis factor-α (TNF-α), interleukin-1β (IL-1β), and interleukin-6 (IL-6), transforming microglia into M2 type and then inhibiting neuroinflammation, partly by weakening the effect of tau kinase, that is, reducing the activity of Tau kinase GSK3β and CDK5 (the activity of PP2A remains unchanged) and reducing the phosphorylation level of Tau, thereby alleviating Tau pathology and playing a neuroprotective effect (Jiang et al., 2016). Similarly, in a mouse model of TREM2 haploinsufficiency, TREM2 deficiency can exacerbate Tau pathology (Sayed et al., 2018). However, it has also been shown that TREM2 deficiency attenuates neuroinflammation and prevents neurodegeneration in a mouse model of tauopathies (Leyns et al., 2017). Additionally, long-term chronic activation of TREM2 may exacerbate Aβ-induced Tau pathology (Jain et al., 2023).

Although it has been generally understood that TREM2 plays an important role in the pathogenesis of AD, the relationship between TREM2 and the phosphorylation of Tau protein remains controversial. Recent studies investigating the effects of activating antibodies against TREM2 in AD have encountered obstacles (Jain et al., 2023; van Lengerich et al., 2023). Therefore, there is an urgent need to explore the specific mechanisms linking TREM2 and Tau in AD.

## 2 TREM2 and it’s main ligands

TREM2 is comprised of three parts: the extracellular domain, transmembrane domain, and intracellular domain (Qin et al., 2021). Among these domains, the extracellular domain can bind to related ligands. TREM2 has a variety of ligands, mainly free negatively charged molecules bound to the plasma membrane (Kober and Brett, 2017), such as heat shock protein 60 (Hsp60) (Stefano et al., 2009), ApoE (Atagi et al., 2015), Aβ (Zhao et al., 2018), galectin-3 (gal3) (Boza-Serrano et al., 2019), sphingosine-1-phosphate (S1P) (Xue et al., 2022) and TDP-43 (Xie et al., 2022), among others (Table 1). Recent studies have also shown that TREM2 can prevent complement-mediated synapse loss by binding to complement C1q (Zhong et al., 2023). The TREM2-APOE pathway is an important mediator in regulating the functional phenotype of microglia, and TREM2 deficiency may lock microglia in homeostasis and hinder the defense function of microglia (Krasemann et al., 2017). Disease-associated microglia (DAM) activation occurs in two stages, the first stage is TREM2-independent, and the second stage is TREM2-dependent. This finding supports that the loss of Trem2 in microglia in the late stage of AD but not in the early stage will aggravate the disease manifestations, which has certain significance for grasping the timing of AD treatment (Keren-Shaul et al., 2017). In addition, another study has found that in non-demented individuals at risk for AD, higher concentrations of disease-associated microglia stage 2 (DAM2) are associated with reduced tau aggregation and alleviated cognitive decline, indicating that activation of microglia to DAM2 can delay the progression of AD (Pereira et al., 2022).

**TABLE 1:** TREM2 and it’s main ligands.

Ligand	Main results	Ref
Hsp60	Binding of TREM2 to Hsp60 exposed at the surface of cells closely interacting with microglia	Stefano et al. (2009)
ApoE	ApoE-TREM2 interaction in microglia plays critical roles in modulating phagocytosis of ApoE-bound apoptotic neurons	Atagi et al. (2015)
Aβ	TREM2 as a microglial Aβ receptor transducing physiological and AD-related pathological effects associated with Aβ	Zhao et al. (2018)
gal3	Galectin-3, a novel endogenous TREM2 ligand, detrimentally regulates inflammatory response in AD	Boza-Serrano et al. (2019)
S1P	Sphingosine-1-phosphate, a novel TREM2 ligand, promotes microglial phagocytosis to protect against ischemic brain injury	Xue et al. (2022)
TDP-43	TREM2 interacts with TDP-43 and mediates microglial neuroprotection against TDP-43-related neurodegeneration	Xie et al. (2022)
C1q	TREM2 receptor protects against complement-mediated synaptic loss by binding to complement C1q during neurodegeneration	Zhong et al. (2023)

Most of these ligands are markers of tissue damage. In the physiological state, the activity of TREM2 is limited to specific tissues, while in the pathological state, the TREM2 signaling pathway becomes an important immune signaling hub for sensing tissue damage (Deczkowska et al., 2020).

After binding to the ligand, TREM2 mainly recruits tyrosine-protein kinase SYK and phosphatidylinositol 3-kinase (PI3K) through its intracellular adaptors DAP12 and DAP10, respectively (DAP12 to SYK, DAP10 to PI3K). This transmits signals into the cell to enable microglia to play roles related to proliferation, phagocytosis, and inflammation (Ulland and Colonna, 2018).

## 3 TREM2 is a potential AD biomarker

Because TREM2 can recognize a variety of ligands closely related to AD and is one of the key regulators of microglial phenotype switching, it plays an important role in the progression of neuroinflammation (Tamburini et al., 2023). Therefore, TREM2 has been extensively studied as a potential AD biomarker. Some studies have found that TREM2 promotes non-inflammatory neuron phagocytosis (Wang and Weaver, 2022), which is different from others who believe that TREM2 acts on neuroinflammation by regulating microglial activation. The full-length TREM2 protein will be cleaved by a disintegrin and metalloproteinase (ADAM), among others, to produce soluble TREM2 (sTREM2). AD variants, specifically rs7922621, are potent variants among other designated variants that control the expression of TSPAN14 (which promotes ADAM10 maturation and trafficking to the cell surface) in the same Candidate cis-regulatory elements (cCREs), exhibiting reduced ADAM10 on the microglia surface and shedding of sTREM2. H1-differentiated microglia-like cells with the rs7922621 risk allele (A/C) had lower levels of cell surface ADAM10 compared with isogenic microglia homozygous for the non-risk allele (Yang et al., 2023). sTREM2 may be used as a bait receptor to competitively bind with the TREM2 ligand, which can weaken the effect of TREM2, thus leading to nerve injury or protection (Piccio et al., 2008; Zhong et al., 2017). Although the endogenous function of sTREM2 itself is not very clear, it serves as a marker of the TREM2 signaling pathway (Morenas-Rodríguez et al., 2022). sTREM2 can be detected in the CSF in healthy individuals and AD patients (Carmona et al., 2018), while the level of sTREM2 in the CSF of AD patients is higher (Yang et al., 2020). Existing studies show that this association with AD is reflected in the CSF rather than plasma (Piccio et al., 2016). sTREM2 in plasma may be associated with other diseases, such as white matter lesions (Tsai et al., 2021). Although sTREM2 can serve as a potential biomarker for AD, sTREM2 levels alone may not be sufficient for an accurate diagnosis of AD, and sTREM2 in CSF is complexly associated with other AD biomarkers (Tamburini et al., 2023). Studies have shown that the levels of sTREM2 are positively correlated with the levels of classical CSF markers total Tau (t-Tau) and phosphorylated-Tau (p-Tau), but not with the concentration of CSF Aβ_42_ (Heslegrave et al., 2016; Piccio et al., 2016). Furthermore, individuals with different *TREM2* gene variants also have different levels of sTREM2 in the CSF. Individuals with variants associated with autosomal recessive early-onset dementia show lower levels of CSF sTREM2. In contrast, R47H carriers have significantly higher sTREM2 levels in the CSF than non-carriers (Piccio et al., 2016).

Additionally, it's worth noting that there is a certain relationship between sTREM2 and the progression of AD. Studies show that very early Aβ seeding triggers the production of sTREM2 even before amyloid PET imaging detects any Aβ plaque deposition (Morenas-Rodríguez et al., 2022). This may subsequently manifest as inflammatory hyperglucose metabolism and may contribute to subsequent increases in p-Tau181 in the earliest stages of AD (Biel et al., 2023). Furthermore, sTREM2 may serve as a potential predictive biomarker for the conversion of mild cognitive impairment (MCI) to AD (Zhao et al., 2022a).

## 4 Effects of TREM2 mutations and altered expression on Tau phosphorylation

TREM2 and microglia play important roles in limiting the development of Tau pathology around plaques. Studies have shown that reduced TREM2 signaling can decrease the response of microglia to pathological Tau (Lee et al., 2021b), and to some extent, reduce the ability of microglia to promote Tau diffusion (Lee-Gosselin et al., 2023). However, it has also been shown that the loss of Trem2 can enhance Tau diffusion through microglial exosomes (Zhu et al., 2022). Moreover, partial or normal function of TREM2 can cause Tau disease and Tau-mediated damage, whereas complete loss of function can reduce Tau-mediated brain damage (Gratuze et al., 2018). Similar to the effect of the TREM2 R47H variant, TREM2 deletion can also prevent P301S mice from atrophy and reduce inflammation, while in the Tau TREM2 haploinsufficiency mouse model, TREM2 deficiency can exacerbate Tau pathology (Jiang et al., 2015; Sayed et al., 2018). However, in the presence of Aβ pathology, the absence of TREM2 will further aggravate the accumulation and spread of Tau, and promote brain atrophy. This effect may be related to the process of TREM2 reducing Aβ itself to promote Tau pathology (Lee et al., 2021a). Notably, there is a complex interplay between ApoE4 and TREM2, and TREM2 deficiency further exacerbates neurodegeneration in Tau mutant mice expressing human ApoE4 (Gratuze et al., 2023). Additionally, TREM2 loss-of-function increases amyloid seeding but reduces plaque-associated ApoE (Parhizkar et al., 2019).

Mutations and polymorphisms of the *TREM2* gene are associated with a significant increase in the risk of AD (Jonsson et al., 2013; Cuyvers and Sleegers, 2016), but it will not increase the risk of Amyotrophic Lateral Sclerosis (ALS) and PD (Zhang et al., 2020). Among the various variants, the R47H variant is particularly noteworthy (Sayed et al., 2021) Moreover, experiments have found that the CSF sTREM2 level in carriers of R47H variants is significantly higher than that in non-carriers (Deming et al., 2019). The concentration of t-Tau and p-Tau in the CSF of patients with p.Arg47His was significantly higher than that in patients without p.Arg47His (Carmona et al., 2018). Additionally, silencing TREM2 in the brains of P301S mice significantly increases the activity of GSK-3β and CDK5, both of which are important factors in Tau hyperphosphorylation (Ballatore et al., 2007; Jiang et al., 2016; Singh et al., 2019).

These findings emphasize the crucial role of TREM2 in the pathogenesis of AD, especially its complex interplay with Tau, which is extensively discussed and studied in relation to AD. The development of anti-AD drugs targeting TREM2 warrants attention and further investigation.

### 4.1 TREM2 variants

TREM2 variants are risk factors for AD and other neurodegenerative diseases (NDDs), and diverse type of TREM2 variants are associated with different NDD risk (Jay et al., 2017b). Variants such as R47H, R62H, or H157Y are more susceptible to AD (Zhou et al., 2019; Li et al., 2021). The most prominent and well-studied of these is the R47H variant, which has been shown to increase AD risk nearly threefold (Guerreiro et al., 2013). The R47H variant is associated with a reduction in the number of receptors, loss of function, and potentially an earlier onset of AD (Li et al., 2021). However, it's contradictory that another study found that the R47H variant was strongly associated with late-onset AD, showing an effect size similar to that of ApoE4 in *Drosophila melanogaster* (Sekiya et al., 2018; Hashioka et al., 2020). Furthermore, the R47H variant may exert neuroprotective effects by reducing brain atrophy, synapse loss, Tau phosphorylation, microglial activation, and phagocytosis of postsynaptic elements in P301S mice. Impaired TREM2 signaling reduces microglia-mediated neurodegeneration in tauopathies (Gratuze et al., 2020). Interestingly, the knockout of TREM2 or TREM2 R47H in APP/PS1 mice reduced microglial proliferation around Aβ plaques and promoted Tau seeding and spreading. These results suggest that TREM2 and its mutations exhibit distinct effects on Tau in the presence and absence of Aβ (Leyns et al., 2019).

**FIGURE 1:**
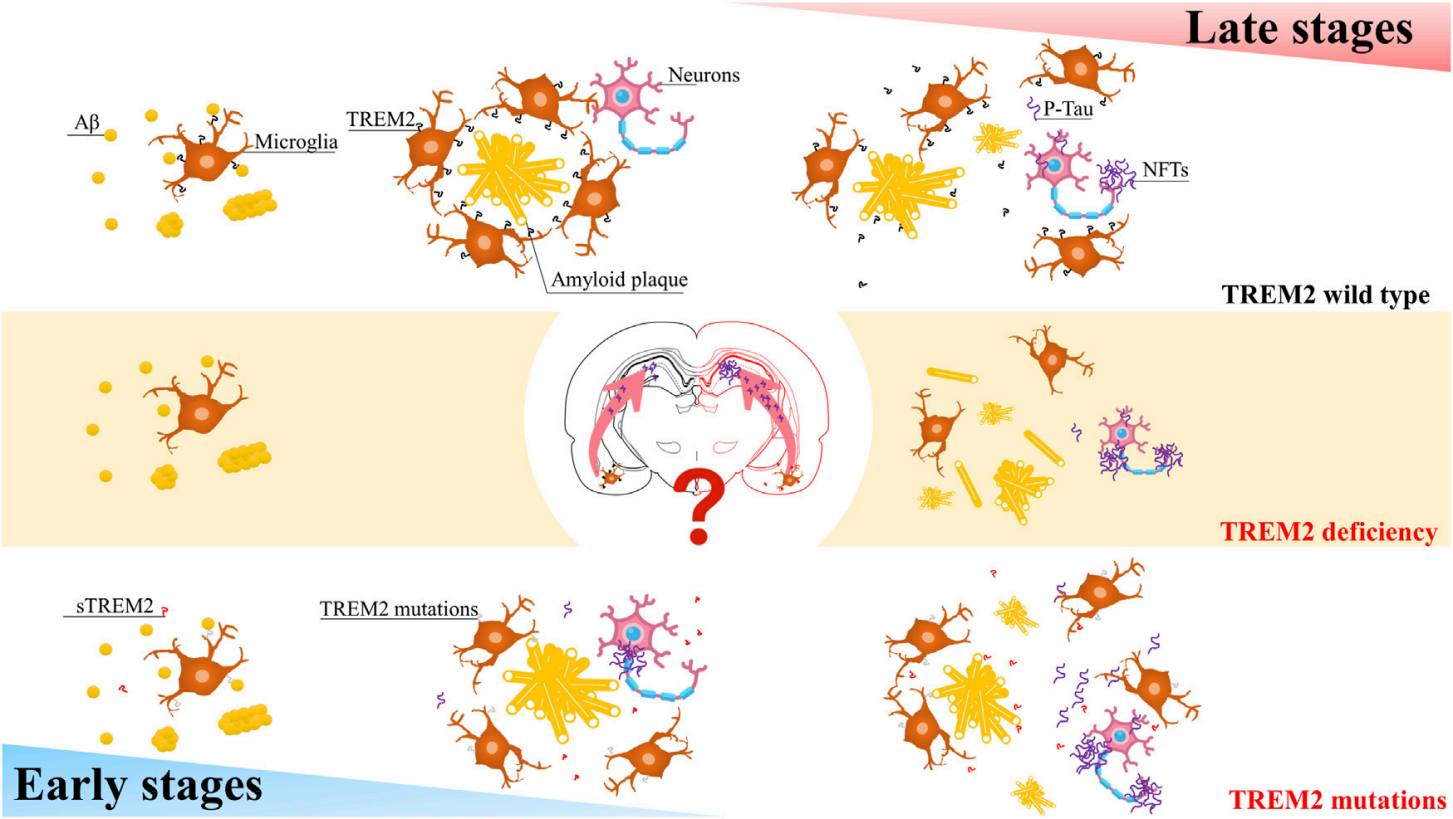
A simplified schematic diagram representing in the different stages of AD, TREM2 have varying effects on Tau pathology.

The role of TREM2 may depend on Aβ pathology and the stage of the disease (Figure 1). TREM2 lies at the critical intersection of Aβ and Tau pathology (Leyns et al., 2019). In the early stages, the R47H variant can reduce the proliferation of microglia around senile plaques, thereby increasing their numbers and promoting the spread of Tau (Gratuze et al., 2020; Perea et al., 2020). In contrast, in the advanced later stages of the disease, when Tau pathology is evident, this variant attenuates the loss of Tau-dependent synapses by reducing the phagocytosis of microglia (Gratuze et al., 2020; Perea et al., 2020). Does this mean that in the early stages of AD, when Tau pathology is not as evident, the R47H variant reduces the maturation of TREM2, preventing the shedding of ADAM protease, which creates more sTREM2? A large amount of sTREM2 weakens the phagocytic activity of cells expressing TREM2, thereby reducing microglial expression. As AD progresses, the microglial barrier around neurofibrillary tangles (NFTs) breaks, releasing Tau and promoting its spread. Furthermore, the increasing amount of Tau will decrease the phagocytic activity of microglia, further exacerbating the spread of Tau. Additionally, the decrease in TREM2 will activate GSK-3β and CDK5, generating more p-Tau and forming a vicious cycle.

### 4.2 Expression of TREM2 and dual roles of TREM2 in AD

In some research related to TREM2 and microglia in AD, microglia and TREM2 appear to play contradictory roles in the pathogenesis of AD. Microglia, like a double-edged sword in AD, on the one hand, gather around NFTs in the brains of AD patients, engulf Aβ (Hickman et al., 2008), Tau (Luo et al., 2015), and abnormal synapses (Hansen et al., 2018), thereby exerting a neuroprotective effect. On the other hand, they produce proinflammatory mediators such as IL-1β, IL-6, and TNF-α, contributing to neurodegenerative changes in AD (Griffin et al., 1998; Paganelli et al., 2002; Uslu et al., 2012). Moreover, treatment strategies that regulate microglial metabolism programs have demonstrated neuroprotective effects, reducing amyloid and Tau load, and improving cognitive deficits (Fairley et al., 2021). As for TREM2, the role of TREM2 in tau pathology in early and late stages of AD disease seems contradictory what have mentioned previously. Well, study shows that some effects of TREM2 on Aβ pathology may be disease-stage-dependent, too (Jay et al., 2017a). Additionally, sTREM2 levels are elevated in the early stages of AD patients' CSF, and these levels are positively correlated with the levels of classic CSF markers t-Tau and p-Tau (Yang et al., 2020), but are unrelated to Aβ or ApoE4 status or gender (Knapskog et al., 2020).

Similarly, the expression of TREM2 also plays a controversial role in the development of AD. AD mice deficient in TREM2 showed both an increase (Wang et al., 2015) and a decrease (Jay et al., 2015) in the number of hyperphosphorylated Tau markers around the plaque. In APP/PS1 mice, upregulation of TREM2 can inhibit GSK-3β, the major kinase involved in Tau hyperphosphorylation in AD, by activating the PI3K/Akt signaling pathway, thereby inhibiting the phosphorylation of Tau protein, and has similar effects in SH-SY5Y cells (Peng et al., 2023). In BV2 cells, TREM2 can inhibit LPS-mediated neuronal apoptosis by downregulating inducible iNOS (M1) and upregulating Arg-1 expression in BV2 microglia (Ni et al., 2022). Another study found that LP17, the synthetic peptide blocker of TREM1, inhibited 6-hydroxydopamine-induced locomotor defects and iNOS messenger RNA expression in rat and zebrafish PD models (Feng et al., 2019). In terms of activating TREM2 to restore microglial activity, AL002 is a humanized monoclonal IgG1 antibody that can bind to the TREM2 microglial receptor and activate signaling, increase the phosphorylation of the TREM2 downstream effector Syk, and induce microglia. Glial cell proliferation. In in vivo studies, activation of TREM2 by AL002 reverses amyloid, promotes microglia recruitment, and improves neurological function (Wang and Weaver, 2022). In a TBI mouse model established by controlled cortical shock (CCI), the TREM2 agonist COG1410 alleviated neural damage by activating the Akt/CREB/BDNF signaling axis in microglia after CCI, and improved the neurobehavior and neurobehavior of mice after CCI Brain electrophysiological activity (Yan et al., 2022). Similarly, activating TREM2 can improve neurological function after intracerebral-hemorrhage (ICH) and reduce neuroinflammation and neuronal apoptosis through the GSK-3β-PI3K/Akt pathway (Chen et al., 2020). In aged TgF344-AD rats, levels of phosphorylated Tau and the Tau kinase Akt3 were significantly increased, while TREM2 was reduced (Bac et al., 2023).

TREM2 exhibits varying effects—protective or nerve-damaging—depending on the context. It may also damage neurons in the following ways: 1. The TREM2 R47H variant promotes the seeding and spreading of Tau aggregates in nerve plaques (Wang et al., 2016). 2. TREM2 deficiency prevents microglia from aggregating around Aβ deposits, causing senile plaques to spread more and thus increasing neuronal damage (Griciuc and Tanzi, 2021). This raises questions about the diversity of functions of TREM2 and microglia, which may be closely related to the TREM2-DAP12 pathway, TREM2 mutations, and other factors. Regional specificity of TREM2 expression in a *postmortem* analysis of primarily non-Hispanic whites, where cortical TREM2 levels were positively associated with AD diagnosis, cognitive decline, and amyloid beta neuropathology, and caudal TREM2 Levels are inversely related to AD neuropathology, indicating that the association of trem2 with Tau burden may depend on disease status (Winfree et al., 2023).

There is also controversy surrounding targeting TREM2 for AD treatment. Studies have shown that TREM2-activating antibodies have favorable effects in enhancing microglial migration and phagocytosis towards amyloid plaques, reducing endogenous Tau hyperphosphorylation, and improving cognitive function (Zhao et al., 2022b). However, it has also been shown that sustained activation of microglia via TREM2, without robust amyloid removal, may exacerbate Aβ-induced Tau pathology (Jain et al., 2023). Therefore, we should exercise caution in considering the activation or inhibition of TREM2 in research and the development of anti-AD drugs.

## 5 TREM2 and other neurodegenerative diseases

Besides its strong association with AD, TREM2 also exerts a significant impact on other neurodegenerative conditions. Since the earliest discovery that the DAP12/TREM2 signaling pathway in human microglia and osteoclasts is associated with NHD (Bianchin et al., 2004; Kaneko et al., 2010). It has been found that TREM2 mutations not only a risk factor for AD, but also for FTD, PD, and ALS (Ogonowski et al., 2023), but so far, no definite conclusion has been drawn. Frontotemporal dementia, an insidious neurodegenerative clinical syndrome, is a common type of dementia characterized by progressive impairment of behavior, executive function, and language (Bang et al., 2015). In a meta-analysis, rs75932628 was confirmed to be associated with FTD susceptibility, while, it was not significantly associated with PD when not divided into races, but in distinguishing North American and European subgroups rs75932628 was significantly associated with PD risk in North America. However, the relationship between SNP and Europeans was not statistically significant, but the meta-analysis included limited samples, which may have certain limitations (Zhou et al., 2019). In 2013, a correlation between p. Arg47His and Parkinson’s disease risk was identified (Bird, 2013). In the TREM2 research analysis of PD blood and CSF, it was found that tau protein positive group levels were associated with sTREM2. Another study found that sTREM2 in CSF had no significant difference between the healthy group and PD patients, but it had a certain effect on predicting cognitive decline in PD. So, they speculated that CSF sTREM2 might be a promising predictor of cognitive decline in PD, but not a diagnostic biomarker (Qin et al., 2022). Despite numerous studies suggesting that TREM2 plays a significant role in the pathogenesis of neurodegenerative diseases, the exact mechanisms and causative relationships between TREM2 and these diseases remain unclear. As a result, further in-depth research is urgently needed to fully understand the biological processes involved in TREM2-related neurodegeneration.

## 6 Conclusion

Over time, studies have focused on the roles of TREM2 variants in regulating microglial responses to Aβ deposition and Tau pathology. However, there are still some shortcomings in current research on TREM2 in the pathogenesis of AD, including: 1. Limited understanding of the underlying mechanisms: although TREM2 has been identified as an important player in the pathogenesis of AD, the exact mechanisms by which it contributes to the disease process are not yet fully understood. 2. Lack of clear causal relationships: although TREM2 has been shown to be associated with an increased risk of AD, it is not yet clear whether this association is causal or simply a marker of disease severity. 3. Variability in findings: some studies have reported conflicting results regarding the role of TREM2 in AD, making it difficult to draw definitive conclusions. 4. Limited clinical implications: despite the promising findings on TREM2, there are currently no approved therapeutic agents targeting this protein, limiting its clinical usefulness.

In our review, we concluded that the effects of Tau pathology and TREM2 on AD and their recent links may provide directions for future treatment-related targets and research. For the future, many questions remain that require further investigation, such as: 1. Is it possible to reduce the production of sTREM2 by promoting the shedding of ADAM, thereby reducing Tau seeding and spreading? 2. Given the complexity of the role of TREM2 in Tau, choosing the appropriate subtype or indication for the development of drugs targeting TREM2 for AD treatment will be a significant challenge. Addressing these issues will enhance our understanding of the TREM2 signaling pathway in AD and aid in the development of new treatment strategies. More studies with adequate follow-up are needed to further evaluate these findings and elucidate the underlying mechanisms.
